# Association Between Excessive Internet Use Time, Internet Addiction, and Physical-Mental Multimorbidity Among Chinese Adolescents: Cross-Sectional Study

**DOI:** 10.2196/69210

**Published:** 2025-05-21

**Authors:** Huiwen Gu, Bing Shi, Huanying He, Sumei Yuan, Jijiao Cai, Xiaofang Chen, Zhongxiao Wan

**Affiliations:** 1 Department of Nutrition and Food Hygiene, School of Public Health Suzhou Medical College of Soochow University Suzhou China; 2 Suzhou Industrial Park Center for Disease Control and Prevention Suzhou China

**Keywords:** physical-mental multimorbidity, internet use, mediation analysis, adolescent, cross-sectional study

## Abstract

**Background:**

In contemporary society, the lives of adolescents are profoundly influenced by the internet. While irrational internet use may have an impact on the physical and mental well-being of teenagers, the relationship between excessive internet use and physical-mental multimorbidity in adolescents remains unclear.

**Objective:**

The aim of this study was to examine the relationship between excessive internet use and physical-mental multimorbidity among adolescents in China.

**Methods:**

A total of 5842 students aged 13 to 18 years from Suzhou city in Eastern China were recruited. Four specific physical disorders and a mental disorder were considered to assess the physical-mental multimorbidity, that is, obesity, hypertension, myopia, dental caries, and depressive symptoms. Logistic regression models were used to evaluate the odds ratios (ORs) and 95% CIs between internet use time, internet addiction (IA) behaviors, and physical-mental multimorbidity. Mediation analyses were performed to explore the mediating effect of sleep duration, diet scores, and tobacco or alcohol consumption on the association between excessive internet use and physical-mental multimorbidity.

**Results:**

A total of 973 (16.7%) students exhibited physical-mental multimorbidity. Students with excessive internet use time (≥2 hours per day) were associated with 45% higher odds of physical-mental multimorbidity compared to their peers who reported internet use for <1 hour per day. Among children and adolescents, a significant J-shaped association was observed between internet use time and physical-mental multimorbidity (nonlinear *P*<.001). Diet score (16.3%) and tobacco or alcohol consumption (12.7%) partially mediated the relationship. Students who met 1 IA behavior (OR 2.44, 95% CI 2.00-2.98) or ≥2 IA behaviors (OR 5.80, 95% CI 4.90-6.86) were associated with higher odds of physical-mental multimorbidity. In the total population, a positive nonlinear correlation was identified between the number of IA behaviors and physical-mental multimorbidity (nonlinear *P*<.001). Sleep duration (2.3%), dietary scores (6.1%), and tobacco or alcohol consumption (6.2%) partially mediated the association.

**Conclusions:**

Excessive internet use is associated with increased odds of physical-mental multimorbidity among adolescents. Sleep duration, dietary quality, and tobacco or alcohol consumption may partially mediate this relationship. These findings highlight the need for monitoring and promoting healthy internet habits as well as addressing lifestyle factors in order to prevent and control physical-mental multimorbidity among adolescents. This research will also provide references for managing internet use and physical-mental health as well as for future longitudinal studies.

## Introduction

The concept of physical-mental multimorbidity, defined as the co-occurrence of at least 1 physical illness alongside at least 1 mental illness, is increasingly garnering attention [1,2]. Globally, 15% of children and adolescents are affected by physical-mental multimorbidity [1]. China is experiencing a significant transition in the spectrum of diseases from infectious to noncommunicable diseases even for children and adolescents [3,4]. Moreover, the impact of mental illness on disease burden cannot be underestimated. The highest disability-adjusted life years for noncommunicable diseases among Chinese adolescents are primarily associated with mental disorders, with depression being a significant component [5]. The presence of physical-mental multimorbidity in children and adolescents is associated with more severe symptoms and greater functional impairment in comparison to peers who are affected by only a single physical or mental condition [6]. Furthermore, given that the majority of adult mental disorders manifest during childhood or adolescence [7], it is likely that the onset of physical-mental multimorbidity typically occurs early in life. This early onset can have long-term implications for children and adolescents, their families, the health care system, and society as a whole [8]. In particular, cost calculations indicate that a small percentage of children (about 5%, usually multimorbid) account for nearly 60% of health care expenditures for individuals younger than the age of 18 years [9]. Altogether, the interplay of multimorbidity and its subsequent effects represents a considerable challenge to public health.

The rapid advancement of technology has facilitated global access to a diverse array of information via the internet, resulting in a notable increase in internet use, particularly among adolescents [10]. The internet has a mixed effect on the well-being of children and adolescents [11]. While a moderate amount of time spent on the web can yield positive outcomes [12], excessive use and internet addiction (IA), often referred to as problematic internet use, can lead to adverse effects [13]. In previous studies, internet use time of ≥2 hours per day has been associated with excessive internet use. Moreover, excessive internet use is more likely to negatively impact adolescents’ physical and mental health [14,15]. IA is defined as a range of adverse symptoms associated with mental dysfunction and behavioral impairment, in addition to excessive internet use [13,16]. IA is often assessed based on several behavioral addiction criteria, including an inability to control internet use and a diminished interest in other activities. Generally, the number of these IA behaviors present can indicate the severity of IA [17,18]. The rising incidence and detrimental impacts of IA have been emphasized by numerous studies conducted across diverse regions, particularly among adolescents [19,20]. In China, the prevalence of IA is also concerning, with an estimated prevalence rate of approximately 19.5% [21]. Besides, excessive internet use has a negative impact on individuals’ physical and mental health. Cohort studies have demonstrated that excessive internet use increases the risk of obesity [22] and depression [23,24] among adolescents. Many studies have also shown that excessive internet use is strongly associated with hypertension [25], dental caries [26], and myopia [27] in adolescents. In addition, lifestyle factors such as diet and sleep have been shown to mediate the association between excessive internet use and health outcomes in adolescents, according to previous studies [22,26,28]. There is evidence that excessive internet use increases the odds of smoking and consuming alcohol in adolescents [29], which can affect their physical and mental health. Therefore, in our study, we hypothesized that individuals who use the internet excessively are more likely to experience physical-mental multimorbidity and that this association is mediated by sleep duration, diet score, and tobacco or alcohol consumption. However, to the best of our knowledge, this issue has not yet been investigated. Existing research on the impact of excessive internet use on children and adolescents often concentrates on a single disorder, neglecting the importance of physical-mental multimorbidity, which can be more severe. Investigating excessive internet use alongside physical-mental multimorbidity can also help promote the simultaneous prevention of multiple diseases. Furthermore, the participants of existing research are typically college students and adults [14]. Given that school-age children generally exhibit poorer self-control and cognitive abilities compared to college-age individuals or adults, the effects of excessive internet use on the health of this population have significant implications. These effects not only influence their individual physical and mental well-being and development but also extend to China’s capacity for fostering a healthy and productive future population. To address existing knowledge gaps and test our hypothesis, via a cross-sectional study in Suzhou city, Eastern China, this study aimed to investigate the association between internet use and physical-mental multimorbidity and further explore the mediating effect of sleep duration, diet scores, and tobacco or alcohol consumption between the associations among adolescents.

## Methods

### Ethical Considerations

This study has been approved by the ethics committee of Suzhou Industrial Park Center for Disease Control and Prevention (SIPCDC-EA-2024005-01). Informed consent and the ability of participants to opt out have been provided. The data are anonymized. No compensation is provided to participants for this human participant research.

### Study Participants

The foundation of this research is the “China Common Disease and Risk Factor Surveillance Among Students,” an annual health survey conducted in schools aimed at enhancing Chinese students’ physical and mental well-being as well as preventing and controlling common diseases [2,30]. This study used data from this survey conducted in the Suzhou Industrial Park area, Suzhou city, Jiangsu province, between 2019 and 2023. In total, 8 schools were randomly selected, comprising 2 elementary schools, 2 junior high schools, 2 senior high schools, 1 vocational high school, and 1 university. Students in grades 1 and above were recruited using stratified cluster sampling. All students provided written consent to participate in this study and were subjected to physical examinations. For our study, junior and senior high school students completing a questionnaire that encompass content pertaining to IA were considered as eligible participants. After removing participants missing necessary data, 5842 participants were included for the final analysis (Multimedia Appendix 1).

### Assessment of Internet Use

Respondents were requested to report the total time they spent on the web weekly and whether they had engaged in any specific IA behaviors during the last week. A 10-item IA questionnaire derived from an updated Young’s diagnostic questionnaire (Cronbach α=0.805) was used to evaluate the IA symptom severity [13,21]. The scale has a total score range of 0-10, with higher scores representing the presence of more IA behaviors. Based on current studies on the prevalence of IA in Chinese children and adolescents, internet use time was categorized into 3 groups: 0-1, 1-2, and ≥2 hours per day [14,21]. The number of IA behaviors was categorized according to tertiles.

### Ascertainment of Physical-Mental Multimorbidity

Students who experienced 1 or more physical morbidities concurrently with depressive symptoms were categorized as having physical-mental multimorbidity. Based on available medical examination and questionnaire data and the previous study [2], a wide range of adolescent diseases was considered to define physical-mental multimorbidity. Ultimately, physical conditions examined in this study comprised hypertension, obesity, myopia, and dental caries, while mental morbidity was indicated by the presence of depressive symptoms. Trained technicians from nearby community health centers conducted physical examinations in accordance with a national standard protocol. A calibrated electronic sphygmomanometer was used to measure diastolic blood pressure (DBP) and systolic blood pressure (SBP) twice on the right upper arm. If the 2 readings of either DBP or SBP differ by more than 5 mm Hg, a remeasurement is warranted. The interval between each measurement and the last one is 1 to 2 minutes. According to Chinese reference standards [31,32], hypertension was identified based on the average of the measurements, with an SBP or DBP value at or above the 95th percentile specific to sex, age, and height being used as the threshold for diagnosis. BMI was calculated as weight divided by the square of height (kg/m^2^). According to Chinese reference values [33], school-age children and adolescents were considered as obese if their BMI was ≥95th percentile for both age and sex. Eye examinations encompassed assessments of distance visual acuity using a retro-illuminated logarithm of the minimum angle of resolution chart featuring tumbling-E optotypes as well as measurements of sphere and cylinder refractive errors conducted with a table-mounted noncycloplegic autorefractor [34]. The calculation of spherical equivalent refraction entails the summation of sphere power and half of the cylinder power. Myopia was defined as uncorrected visual acuity <5.0 and spherical equivalent refraction <–0.5 diopter [35]. Individuals who exhibit myopia in 1 eye are classified as myopic. Additionally, individuals recognized as wearing orthokeratology lenses are also counted as myopic. Dental caries were detected during an oral examination, and cavities, caries-related tooth loss, and fillings on tooth surfaces were identified.

The assessment of depressive symptoms was conducted using the Chinese version of the Center for Epidemiologic Studies Depression Scale (CES-D), a scale designed to evaluate the severity of depressive symptoms experienced over the preceding week [36]. The CES-D consists of 20 items, which are structured on a 4-point scale ranging from 0 to 3. Higher scores on the CES-D reflect a greater severity of depressive symptoms. The total score can range from 0 to 60, with a score of 16 or higher suggesting the presence of depressive symptoms [36,37]. It has already been proven that CES-D is valid and reliable among the Chinese population [38,39], and in this study, the scale’s Cronbach α was 0.879.

### Assessment of Covariates

We included age, sex, grade, number of family members, BMI, and physical activity as covariates in the model. Age and sex are traditional confounding factors, and demographic information such as education level and family size to some extent represent the environment of adolescents. BMI and moderate to vigorous physical activity (MVPA) represent physiological indexes and life behavior factors, respectively. They both affect internet use and psychosomatic comorbidities. A validated questionnaire was used to collect demographic data [30,40]. Age was determined by deducting the date of birth from the date of physical examination. Sex was categorized as either boy or girl. Educational attainment was classified as junior high school (grade 7-9) or senior high school (grade 10-12). The number of family members residing in the same household was categorized into ≤2, 3, ≥4. Due to the minimal representation of underweight individuals, they were integrated into the normal BMI category. Consequently, BMI was graded as normal, overweight, and obese according to the reference values for Chinese school-age children [33]. MVPA was evaluated through the following question: “how many days did you participate in MVPA for at least 60 minutes over the past week? MVPA covers any physical activity that elevates heart rate or respiratory rate, such as weightlifting, swimming, basketball, football, running, and aerobic exercises).” Meeting the MVPA recommendation necessitated reporting 7 days of daily MVPA for a minimum duration of 60 minutes [41].

### Assessment of Mediators

Based on previous studies [22,26,28,29], sleep duration, diet score, and tobacco or alcohol consumption were examined as mediating variables because they lie on the inferential pathway from exposure to outcome. Sleep duration was assessed using the following question: “how much sleep do you get on average per day?” According to the 24-hour movement guidelines, children aged 6-13 years should sleep for 9-11 hours, while adolescents aged 14-17 years should sleep for 8-10 hours. Students who reported the recommended sleep duration were deemed to be in compliance with the recommendations [41]. Diet quality was assessed by a diet score, which is calculated based on the consumption frequency of 5 components (sugary beverages, fried foods, fruits, vegetables, and breakfast) over the preceding 7 days. Each component is assigned a rating ranging from 1 to 3, where higher scores indicate a greater frequency of consumption of fruits, vegetables, and breakfast and a lower frequency of sugary drinks and fried foods, thereby reflecting healthier dietary habits. Meeting any of the following was considered an unhealthy diet: the intake of sugary beverages or fried foods ≥1 time per day, eating fruits or vegetables less than 1 time per day, and the occasional or daily omission of breakfast [42]. The act of smoking cigarettes, having smoked cigarettes within the past 30 days, or consuming a full glass of alcohol is classified as tobacco or alcohol consumption.

### Statistical Analysis

All continuous variables included in the analysis were reported as medians along with IQRs, as they exhibited skewness in normality tests. Categorical variables were represented using frequencies and relative percentages (%). The chi-square test was used for categorical variables, while the Wilcoxon rank sum test was used for continuous variables to evaluate differences in characteristics based on the presence or absence of physical-mental multimorbidity among students.

Due to the use of a stratified cluster sampling method, intraclass correlation coefficients for the school and class levels were calculated, yielding values of 0.032 and 0.055, respectively. According to Cohen [43], such small intraclass correlation coefficients indicate that the impact of the nested data structure is minimal. Therefore, a simple model was chosen instead of a multilevel model. Logistic regression models were used to examine the association between internet use time and the number of IA behaviors as categorical variables, in relation to the risk of physical-mental multimorbidity. The results were displayed as odds ratios (ORs) with 95% CIs. Models were adjusted for age, sex, grade, number of family members, BMI, and physical activity. A restricted cubic spline with 5 knots at the quintiles of internet use time or number of IA behaviors was performed to explore the potential dose-response relationship between internet use time and the number of IA behaviors associated with the risk of physical-mental multimorbidity. Subgroup and interaction analyses were conducted on participants with varying baseline characteristics to further explore the potential effect modifiers. Mediation hypotheses of sleep duration, dietary scores, and tobacco or alcohol consumption on the relationship between excessive internet use and physical-mental multimorbidity were evaluated using the bias-corrected bootstrap method with 5000 iterations to determine average causal mediation effects (indirect effect; ACMEs), average direct effects, and mediating proportion and obtain 95% CIs, considering an effect significant if the 95% CI did not include 0. The 3 mediating variables were incorporated into the model individually, with sleep duration and diet score treated as continuous variables, while tobacco or alcohol consumption was classified as a categorical variable. Before conducting the mediation analysis, interactions between the 3 mediator variables and the 2 exposure variables were examined individually. Among these, only the interaction between IA behaviors and sleep duration was found to be significant. Consequently, this interaction term was included in the mediation model to accurately capture the potential moderating effect on the mediation pathway. All statistical analyses were performed using STATA (version 18.0; Stata Corp LLC) and R software (version 4.4.1; R Foundation for Statistical Computing). Statistical significance was defined as a 2-tailed *P* value of <.05.

## Results

### Basic Characteristics of the Participants

The basic characteristics of the 5842 participants are presented in Table 1. The median age of the students was 15.4 (IQR 14.0-16.8) years. Among the participants, 2957 (50.6%) were boys, and 2102 (36%) lived with 0-2 family members. Junior high school and senior high school students comprised 38.9% (n=2272) and 61.1% (n=3570), respectively. Most of them had a normal BMI rating (n=4034, 69%), and a substantial proportion did not meet the recommended standards for physical activity (n=5133, 87.9%). Their median diet score was 13 (IQR 12-13), and median sleep duration was 7.0 (IQR 6.5-8.0). Nearly half (n=2671, 45.7%) of the students use the internet for less than 1 hour per day, and 3835 (65.6%) participants did not exhibit IA behaviors. Additionally, 973 (16.7%) students were identified as having physical-mental multimorbidity. Compared with peers without physical-mental multimorbidity, students with physical-mental multimorbidity tended to be slightly older, girls, in senior high school, and not meet the physical activity and sleep duration recommendation; had a lower diet score; had a higher chance of tobacco or alcohol consumption; allocated more time to web-based activities; and engaged in more IA behaviors (*P*<.05).

**Table 1:** Characteristics of study participants stratified by physical-mental multimorbidity status.

Characteristics			Overall (N=5842)		Physical-mental multimorbidity					*P* value	
					No (n=4869)		Yes (n=973)				
Age (years), median (IQR)			15.4 (14.0-16.8)		15.2 (13.8-16.6)		16.0 (14.8-17.0)		<.001		
**Sex, n (%)**											.01
	Boy	2957 (50.6)		2500 (51.4)		457 (47)					
	Girl	2885 (49.4)		2369 (48.7)		516 (53)					
**Family members, n (%)**											.38
	0-2	2102 (36)		1736 (35.7)		366 (37.6)					
	3	1869 (32)		1557 (32)		312 (32.1)					
	≥4	1871 (32)		1576 (32.4)		295 (30.3)					
**Grade, n (%)**											<.001
	Middle school (grades 7-9)	2272 (38.9)		2021 (41.5)		251 (25.8)					
	High school (grades 10-12)	3570 (61.1)		2848 (58.5)		722 (74.2)					
**BMI (kg/m^2^), n (%)**											.41
	Normal	4034 (69)		3350 (68.8)		684 (70.3)					
	Overweight	1067 (18.3)		904 (18.6)		163 (16.8)					
	Obesity	741 (12.7)		615 (12.6)		126 (13)					
**Physical activity, n (%)**											.04
	Not meet recommendation	5133 (87.9)		4259 (87.5)		874 (89.8)					
	Meet recommendation	709 (12.1)		610 (12.5)		99 (10.2)					
Diet score, median (IQR)			13 (12-13)		13 (12-13)		12 (11-13)		<.001		
**Diet, n (%)**											<.001
	Healthy	3287 (56.3)		2902 (59.6)		385 (39.6)					
	Unhealthy	2555 (43.7)		1967 (40.4)		588 (60.4)					
Sleep duration (hours), median (IQR)			7.0 (6.5-8.0)		7.0 (6.5-8.0)		6.5 (6.0-7.3)		<.001		
**Sleep duration, n (%)**											<.001
	Not meet recommendation	4651 (79.6)		3800 (78)		851 (87.5)					
	Meet recommendation	1191 (20.4)		1069 (22)		122 (12.5)					
**Tobacco or alcohol consumption, n (%)**											<.001
	No	4660 (79.8)		4023 (82.6)		637 (65.5)					
	Yes	1182 (20.2)		846 (17.4)		336 (34.5)					
**Internet use time (hours per day), n (%)**											<.001
	<1	2671 (45.7)		2312 (47.5)		359 (36.9)					
	≥1 to<2	1763 (30.2)		1450 (29.8)		313 (32.2)					
	≥2	1408 (24.1)		1107 (22.7)		301 (30.9)					
**Internet addictive behaviors met, n (%)**											<.001
	0	3835 (65.6)		3486 (71.6)		349 (35.9)					
	1	881 (15.1)		695 (14.3)		186 (19.1)					
	≥2	1126 (19.3)		688 (14.1)		438 (45)					

### Association Between Internet Use and Physical-Mental Multimorbidity

Internet use was quantified by internet use time and the number of IA behaviors present. In terms of internet use time, those who reported internet use time of 1-2 hours per day (OR 1.33, 95% CI 1.12-1.57) and those who used the internet for 2 hours or more per day (OR 1.45, 95% CI 1.21-1.73) exhibited a heightened odds of physical-mental multimorbidity when compared to those who used the internet for less than 1 hour per day in the fully adjusted model (Table 2). As for IA behaviors, compared to students who did not engage in IA behaviors, individuals exhibiting 1 or more IA behaviors demonstrated a significantly elevated probability of physical-mental multimorbidity. Their adjusted ORs were 2.44 (95% CI 2.00-2.98) and 5.80 (95% CI 4.90-6.86), respectively.

**Table 2:** Association between internet use and physical-mental multimorbidity.

		Events, n/N (%)	Unadjusted OR^a^ (95% CI)	*P* value	Adjusted^b^ OR (95% CI)	*P* value
**Internet use time (hours per day)**						
	<1	359/2671 (13.4)	1 (reference)	—^c^	1 (reference)	—
	≥1 to <2	313/1763 (17.8)	1.39 (1.18-1.64)	<.001	1.33 (1.12-1.57)	.001
	≥2	301/1408 (21.4)	1.75 (1.48-2.07)	<.001	1.45 (1.21-1.73)	<.001
**Tertiles of internet addictive behaviors met**						
	Tertile 1 (0)	349/3835 (9.1)	1 (reference)	—	1 (reference)	—
	Tertile 2 (1)	186/881 (21.1)	2.67 (2.20-3.25)	<.001	2.44 (2.00-2.98)	<.001
	Tertile 3 (≥2)	438/1126 (38.9)	6.36 (5.40-7.48)	<.001	5.80 (4.90-6.86)	<.001

^a^OR: odds ratio.

^b^Models were adjusted for age, sex, grade, number of family members, BMI, and physical activity.

^c^Not available.

**Figure 1:**
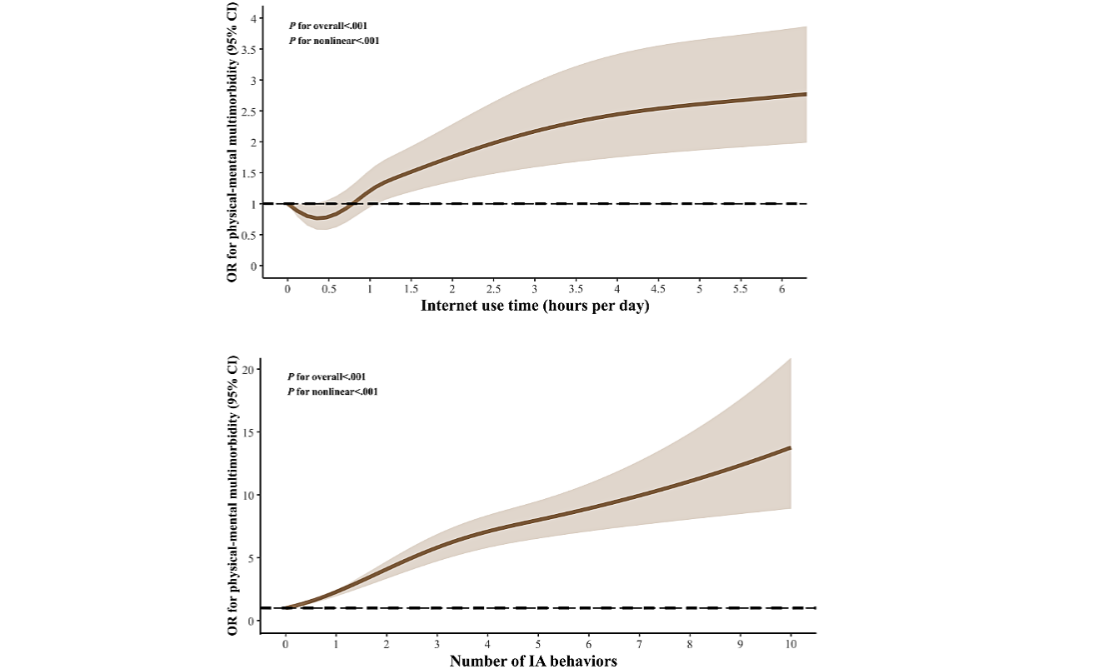
Dose-response association between internet use time and the number of IA behaviors and physical-mental multimorbidity in all students. The dotted line presents the 95% CIs. Models were adjusted for age, sex, grade, the number of family members, BMI, and physical activity. P (nonlinear) was <.001 between internet use time and physical-mental multimorbidity and <.001 between the number of IA behaviors and physical-mental multimorbidity. IA: internet addictive; OR: odds ratio.

Figure 1 demonstrates the dose-response relationship between internet use time and the number of IA behaviors and physical-mental multimorbidity. A notable J-shaped relationship was identified between internet use time and the existence of physical-mental multimorbidity, with a nonlinear *P*<.001. When internet use time exceeded a certain range (about 0.8 hours), it was observed that the odds of physical-mental multimorbidity increased with the increase of internet use time (Figure 1). A dose-response relationship was identified between an increasing number of IA behaviors and a heightened probability of physical-mental multimorbidity (nonlinear *P*<.001). Notably, the rate of increase in odds appeared to plateau for ≥4 IA behaviors (Figure 1).

### Subgroup Analyses

**Figure 2:**
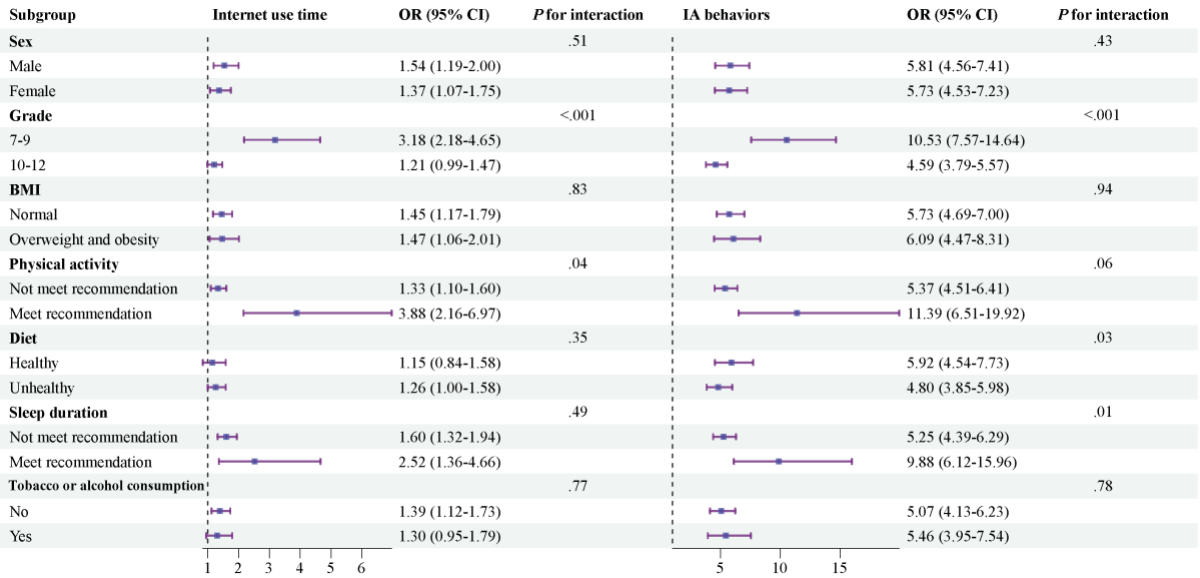
Forest plots for internet use time and the number of IA behaviors in subgroups. ORs and 95% CIs for internet use time ≥2 hours per day and for IA behaviors ≥2 are shown for subgroups as defined by baseline participant characteristics. IA: internet addictive; OR: odds ratio.

As shown in Figure 2, the correlation between internet use time ≥2 hours per day and an increased probability of physical-mental multimorbidity was found to be significantly more pronounced among junior high school students (OR 3.18, 95% CI 2.18-4.65) compared to senior high school students (OR 1.21, 95% CI 0.99-1.47; *P* for interaction<.001) and in individuals who adhered to physical activity guidelines (OR 3.88, 95% CI 2.16-6.97) than in those who did not (OR 1.33, 95% CI 1.10-1.60; *P* for interaction=.04). Nevertheless, sex, BMI classification, diet, sleep duration, and tobacco or alcohol consumption did not significantly influence this association (*P* for interaction>.05).

A similar interaction of grade and physical activity was also seen in the association of IA behavior ≥2 with physical-mental multimorbidity (*P* for interaction<.001 and *P* for interaction=.06). The association was also stronger among junior high school students (OR 10.53, 95% CI 7.57-14.64) and those who adhered to physical activity guidelines (OR 11.39, 95% CI 6.51-19.92). The association between the number of IA behaviors ≥2 and elevated odds of physical-mental multimorbidity was also significantly influenced by diet and sleep duration (*P* for interaction=.03 and .01, respectively). This association was stronger in individuals who adhered to a healthy diet and followed sleep guidelines, with ORs ranging from 5.92 (95% CI 4.54-7.73) to 9.88 (95% CI 6.12-15.96). However, this association was not significantly impacted by sex, BMI classification, and tobacco or alcohol consumption (*P* for interaction>.05).

### Mediating Role of Sleep Duration, Diet Score, and Tobacco or Alcohol Consumption

The total, direct, and indirect effects for the mediating role of sleep duration, diet score, and tobacco or alcohol consumption on the relationship between internet use and physical-mental multimorbidity are presented in Table 3. For internet use time, bootstrapping revealed significant relative indirect effects of diet score (ACME=.007, 95% CI .004-.010) and tobacco or alcohol consumption (ACME=.005, 95% CI .002-.010), indicating that diet score and tobacco or alcohol consumption served as significant mediating variables in the relationship between internet use time and physical-mental multimorbidity, accounting for mediation shares of 16.3% and 12.7%, respectively. Conversely, the mediating effect of sleep duration was found to be nonsignificant. In terms of IA behaviors, sleep duration (ACME=.002, 95% CI .0001-.010), diet scores (ACME=.007, 95% CI .004-.010), and tobacco or alcohol consumption (ACME=.008, 95% CI .004-.010) were found to partially mediate the association between IA behaviors and physical-mental multimorbidity, accounting for 2%, 6.1%, and 6.2%, respectively.

**Table 3:** The mediating effect of sleep duration, diet score, and tobacco or alcohol consumption on the association between internet use and physical-mental multimorbidity^a^.

Bootstrap test		Total effect		ADE^b^		ACME^c^			PM^d^	
		β (95% CI)	*P* value	β (95% CI)	*P* value	β (95% CI)	*P* value	%		*P* value
**Internet use time**										
	Sleep duration	.042 (.022 to .060)	<.001	.045 (.024 to .070)	<.001	–.002 (–.005 to .000)	.06	—^e^		—
	Diet score	.043 (.022 to .060)	<.001	.036 (.015 to .060)	<.001	.007 (.004 to .010)	<.001	16.3		<.001
	Tobacco or alcohol consumption	.042 (.023 to .060)	<.001	.037 (.017 to .060)	<.001	.005 (.002 to .010)	<.001	12.7		<.001
**Number of internet addictive behaviors met**										
	Sleep duration	.120 (.092 to .150)	<.001	.117 (.090 to .150)	<.001	.002 (.0001 to .010)	.04	2.0		.04
	Diet score	.122 (.094 to .150)	<.001	.114 (.087 to .140)	<.001	.007 (.004 to .010)	<.001	6.1		<.001
	Tobacco or alcohol consumption	.121 (.094 to .150)	<.001	.114 (.086 to .140)	<.001	.008 (.004 to .010)	<.001	6.2		<.001

^a^The 95% CI of these estimates was calculated using the bias-corrected bootstrap method (with 5000 iterations). In all mediation analyses, results were adjusted for age, sex, grade, number of family members, BMI, and physical activity.

^b^ADE: average direct effect.

^c^ACME: average causal mediation effect (indirect effect).

^d^PM: mediating proportion, the mediator variable explains the percentage of the association between internet use and physical-mental multimorbidity.

^e^Not available.

## Discussion

### Principal Findings

In our study, we found that both longer internet use time and the manifestation of increased IA behaviors were correlated with higher odds of physical-mental multimorbidity among Chinese adolescents. Mediation analyses demonstrated that sleep duration, dietary scores, and instances of tobacco or alcohol consumption may partially mediate the relationship between internet use time, IA behaviors, and physical-mental multimorbidity.

### Comparison to Prior Work

Excessive internet use time has been demonstrated to be associated with various negative physical health outcomes, including visual impairment, hypertension, and obesity [22,25-27]. In addition, prolonged exposure to internet may result in adolescents isolating themselves from real-life experiences, undermining social support, and potentially leading to psychological issues such as depression [44]. Our study is the very first to demonstrate dose-response positive associations between excessive internet use time and an increased probability of physical-mental multimorbidity in Chinese adolescents. The discovered J-shaped relationship between internet use time and the odds of physical-mental multimorbidity suggests that limited internet use time around <0.8 hours per day might be associated with reduced odds of physical-mental multimorbidity. This can be explained by the positive effects of internet use, which include social interaction, access to support systems, and educational resources [12]. Regarding IA, a significant nonlinear correlation was found between increased IA behaviors and a higher probability of physical-mental multimorbidity. The dose-response curve revealed that adolescents with milder IA manifestations (IA behaviors ≤3) showed a steeper gradient in physical-mental multimorbidity odds, which suggests that early-stage IA in adolescents warrants focused attention. Several studies have highlighted the necessity for early prevention and intervention in IA [45,46].

The observed weaker correlation between internet use and physical-mental multimorbidity among senior high school students, in comparison to junior high school students, may be attributed to the increased cognitive maturity that accompanies age as well as engagement in more meaningful activities [47]. Another explanation is better coping mechanisms and greater resilience in late adolescence [48]. The association between excessive internet use time and elevated odds of physical-mental multimorbidity was stronger among students who engaged in physical activity at recommended levels. A similar trend was observed concerning IA behaviors and physical-mental multimorbidity. Furthermore, increased IA behaviors were associated with significantly higher odds of physical-mental multimorbidity among students who maintained a healthy diet and adhered to recommended sleep durations. This finding suggested that students with healthier lifestyles seem to be more likely to experience physical-mental multimorbidity once they exhibit IA, which appeared to contradict the prevailing assumption. One potential explanation for this phenomenon is that healthier lifestyles exhibited by adolescents may be influenced by parental supervision and the school environment. Parents’ overregulation may harm their children’s mental health [49]. Nevertheless, this attribution is speculative, and further studies are warranted to clarify these associations. Significant mediating effects of sleep duration, dietary scores, and tobacco or alcohol consumption on the relationship between internet use and physical-mental multimorbidity have been observed, although the mediating effect of sleep duration was only found in the relationship between IA behaviors and physical-mental multimorbidity. There exists a well-documented correlation between IA and various sleep-related issues. Excessive internet use can lead to a reduction in sleep duration, an inclination toward late-night activities, the presence of sleep disorders, insomnia, and increased daytime fatigue and lethargy [50]. Statistically, approximately one-third of adolescents keep their cell phones activated during the night [44], and exposure to bright light is associated with the suppression of melatonin production and delayed sleep and wakefulness [50]. Furthermore, sleep disturbances attributed to excessive internet use have been shown to be associated with physical illness, depressive symptoms, and suicidal ideation among adolescents [51]. As for diet, individuals who engage in high-risk internet use may experience an unbalanced nutritional intake and diminished diet quality. This can be attributed to factors such as frequent breakfast skipping, low intake of fruits and vegetables, and excessive snacking [52,53]. Moreover, a study has proved that adolescents with IA are more prone to display selective eating behaviors and show a preference for convenience foods, including fast food and junk food [54]. Consequently, these dietary habits may elevate the risk of health-related conditions. However, some literature also suggests that the internet can promote healthy eating behaviors in adolescents by sharing images and videos of nutritious foods, thereby increasing their interest in and acceptance of healthy eating [55]. This may be attributed to variations in the duration and content of internet use, and further research is necessary to explain this mediating effect. In addition, a significant correlation exists between IA and the excessive consumption of alcohol and tobacco [52,56,57], and the health risks associated with these substances for adolescents are undeniable. Collectively, our findings suggest that unhealthy internet use might be associated with increased odds of physical-mental multimorbidity, with unhealthy lifestyles serving as mediators, particularly sleep, diet score, and tobacco or alcohol consumption. However, this association and mediation do not eliminate the possibility of reverse causation. Further cohort studies on the associations between internet use and physical-mental multimorbidity in adolescents are necessary for consideration.

### Strengths and Limitations

This study possesses several strengths. To the best of our knowledge, it represents the first evaluation of the relationship between excessive internet use and physical-mental multimorbidity among adolescents in an economically developed region of Eastern China. Second, school-age children, a group with special physiological and psychological characteristics, are concerned in this study. However, it is necessary to note that there are certain limitations. First, the cross-sectional design used in this study did not allow for the establishment of a causal relationship between exposure and outcome. Furthermore, the assessment of physical-mental multimorbidity and covariates was constrained by limited data. It may not encompass the full spectrum of physical and mental health conditions and may overlook some potential confounders. Additionally, the sample of participants was exclusively drawn from economically developed regions of China, which may restrict the generalizability of our findings to the broader population of Chinese adolescents. Finally, the data analyzed in this study were obtained from census records of primary care workers rather than from professional researchers, which may introduce some measurement or evaluation bias.

### Conclusions

Excessive internet use time and more IA behaviors were correlated with a heightened probability of physical-mental multimorbidity. Sleep duration, dietary quality, and tobacco or alcohol consumption may serve as partial mediators of this relationship. These findings underscore the importance of monitoring and promoting healthy internet habits among adolescents as well as addressing lifestyle factors that may contribute to their overall well-being. This knowledge will provide valuable insights for regulating adolescent internet use and improving their health outcomes. Future studies are warranted to explore the longitudinal associations between internet use at various developmental stages and the subsequent emergence of physical-mental multimorbidity in adolescents.

## Appendix

Multimedia Appendix 1 Flowchart of the participants.

## Abbreviations

ACME: average causal mediation effect (indirect effect)
CES-D: Center for Epidemiologic Studies Depression Scale
DBP: diastolic blood pressure
IA: internet addiction
MVPA: moderate to vigorous physical activity
OR: odds ratio
SBP: systolic blood pressure

## Data Availability

The datasets used and analyzed during this study are available from the corresponding author on reasonable request.
