# Supplementary figures and images for: Association Between Excessive Internet Use Time, Internet Addiction, and Physical-Mental Multimorbidity Among Chinese Adolescents: Cross-Sectional Study
Source: J Med Internet Res. 2025 May 21;27:e69210. doi: 10.2196/69210 (PMC12138303; doi:10.2196/69210)

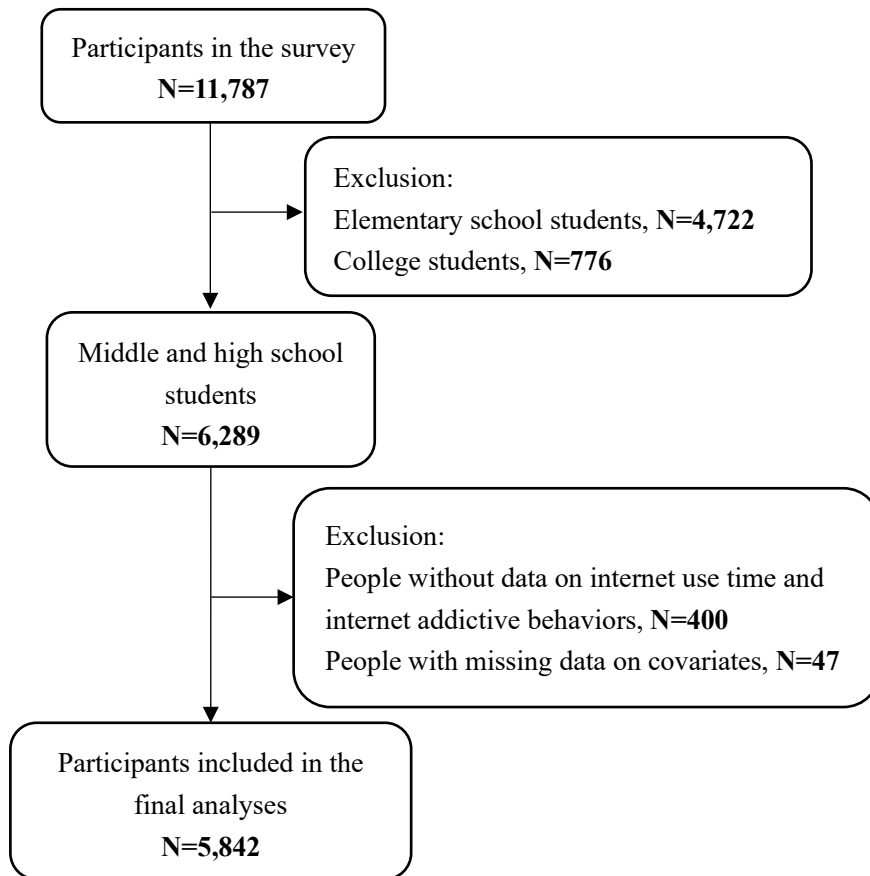

Supplement: Multimedia Appendix 1 [file jmir_v27i1e69210_app1.pdf]
